# Evaluation of Patients Referred for Abnormal Digital Rectal Examination With Normal Prostate-Specific Antigen on Best Timed Pathway for Prostate Cancer

**DOI:** 10.7759/cureus.46012

**Published:** 2023-09-26

**Authors:** Chris B Richards, Alice B Corfield, Paul Cleaveland, Vincent C Tang, Andrew N Sinclair, James E Dyer

**Affiliations:** 1 Urology, Stockport NHS Foundation Trust, Manchester, GBR

**Keywords:** diagnostic pathway, digital rectal examination (dre), prostate-specific antigen (psa), magnetic resonance imaging, prostate neoplasm

## Abstract

Introduction

Currently, there is no recommendation for adjustments to the Best Timed Pathway for Prostate cancer (BTiPP) depending on whether the referral is for raised prostate-specific antigen (PSA) or malignant-feeling prostate on digital rectal examination (DRE). Therefore, all patients undergo MRI scanning. We aim to establish if patients with abnormal DRE only (without raised PSA) should have an adjusted pathway by comparing the biopsy rate and diagnostic yield after an MRI scan.

Methods

All BTiPP 2021 referral patient notes were reviewed. The patients were categorized into the aDRE group (abnormal DRE with normal PSA) or the rPSA group (raised PSA with or without abnormal DRE). Data and results for MRI and prostate biopsy were evaluated. Diagnostic yield was defined as the percentage of patients who underwent an MRI, who were diagnosed with biopsy-proven cancer.

Results

68.5% (74/108) and 70.9% (282/398) of patients underwent upfront MRI in the aDRE and rPSA groups, respectively. Following MRI, the biopsy rate (28.4% (21/74) vs. 42.9% (121/282) (p=0.02)) and the biopsy-proven diagnostic yield (20.3% (15/74) vs. 36.9% (104/282) (p<0.01)) were both significantly lower in the aDRE group. 58% (43/74) of patients in the aDRE group had no posterior lesions on MRI. Only 6.7% (1/15) of biopsy-proven cancers in the aDRE group were solely anterior.

Conclusions

After MRI, the biopsy rate and diagnostic yield were significantly lower in the aDRE group compared to the rPSA group. Furthermore, a majority of patients referred for aDRE had a normal posterior prostate appearance on MRI. An adjusted pathway for patients referred for aDRE with normal PSA, with DRE by a urologist prior to MRI, should be considered as it would likely reduce unnecessary investigations, treatment, and patient anxiety. These data suggest that this would not risk missing significant cancers.

## Introduction

The Best Timed Pathway for Prostate cancer (BTiPP) aims to ensure patients referred with suspected prostate cancer are informed of cancer diagnosis (or exclusion) within 28 days of referral [1]. Patients are referred from primary care with a raised PSA or malignant feeling prostate on digital rectal examination (DRE), then triaged by a urologist or cancer clinical nurse specialist (CNS). Patients with a raised PSA that may be explained by a urinary tract infection (UTI) are triaged off the pathway. Patients are then referred for prostate multi-parametric magnetic resonance imaging (MRI), unless they would be unsuitable for active treatment; have very high PSA indicating metastatic disease; or MRI is contraindicated. They proceed to targeted prostate biopsy if there are suspicious lesions on MRI, indicated by Prostate Imaging-Reporting and Data System score (PI-RADS) >3, or PI-RADS=3 with PSA density (PSAD) >0.15ng/ml2. This pathway is largely based on the PROMIS trial which found that MRI was more sensitive for clinically significant prostate cancer than the previously recommended transrectal ultrasound-guided biopsy [2]. This has led to the recommendation of MRI prior to targeted biopsies.

There is currently no adjustment on the BTiPP for referrals based on whether they are for raised PSA or abnormal DRE. PSA is an objective measure, unlike DRE, which is highly examiner-dependent. A systematic review found that primary care DRE screening was poor for biopsy-proven prostate cancer, with a pooled sensitivity of 51% [3]. Furthermore, half of surveyed primary care physicians in Canada (where DRE forms part of prostate cancer screening) are not confident in DRE, and one-third believe it is not an accurate test [4].

There are multiple studies assessing the value of DRE for prostate cancer investigation in various ways. A large retrospective analysis comparing clinically significant prostate cancer risk between patients with normal and abnormal DRE found that there was more risk with abnormal DRE with increasing PSA, whereas there was no difference when PSA was normal [5]. This was in keeping with the Rotterdam screening study which found that the positive predictive value of DRE increased as PSA increased [6]. The Rotterdam authors also found that cancers detected on DRE with a low PSA were likely to be small and low-grade [6]. One small study of patients with abnormal DRE found that significant cancer detection rate (defined as Gleason grade 3+4=7 or more) was higher with a raised PSA compared to normal PSA (31% vs 5%) [7]. This study also found that concordance between primary and secondary care DRE findings was only 46% [7]. These findings call into question the value of DRE when PSA is normal.

The role of DRE for prostate cancer detection in primary care therefore remains without a clear consensus. The BTiPP suggests that it be included in a “minimum dataset” before specialty referral [1], whereas United States National Cancer Care Network guidelines suggest it only be used for follow-up or an adjunct when PSA is raised [8], and American Urological Association omit it entirely from their guidelines [9].

We aimed to consider whether patients referred from primary care for abnormal DRE only should have an adjusted pathway whereby they are examined by a urologist prior to MRI. The objectives were: following MRI, compare biopsy rate when referred with raised PSA and abnormal DRE alone; compare the combined diagnostic yield of having an MRI and prostate biopsy (if indicated) when referred with raised PSA and abnormal DRE alone; evaluate patients referred with abnormal DRE alone with cancer to determine the cancer location, grade, and treatment; consider with an adjusted BTiPP, how many additional outpatient appointments would be required, and how many MRIs might be avoided.

This article was previously presented as an abstract at the 2023 Royal Society of Medicine Urology Section Meeting on May 12, 2023.

## Materials and methods

Patients referred to our institution on the BTiPP from primary care in 2021 were retrospectively identified from the pathway records. Those with a raised age-adjusted PSA were categorized into an rPSA group (regardless of DRE findings). Raised age-adjusted PSA level was defined as: ≥2.0 ng/ml if ≤49 years; ≥3.0 ng/ml if 50-59 years; ≥4.0 ng/ml if 60-69 years; ≥5.0 ng/ml 70-74 years; ≥7.5 ng/ml if 75-80 years; and as per clinical discretion >80 years. Those with a normal PSA and abnormal DRE were categorized into an aDRE group. Those initially referred for abnormal DRE who subsequently had a raised PSA result were categorised into the rPSA group. Patients referred for other reasons were not categorized into either group and were excluded from the analysis.

Therefore the inclusion criteria were: new patients referred on the BTiPP for either a raised PSA or abnormal DRE. The exclusion criteria were: patients referred on the pathway for another reason; or referred with a raised PSA that was explained by a UTI (proven on urine culture).

The patients’ notes were reviewed to determine whether they underwent MRI or biopsy and if the biopsy resulted in a cancer diagnosis (defined as Gleason grade 3+3=6 or more). The aDRE group patients who were diagnosed with cancer were then analyzed further to determine the following: prostate gland volume on MRI, location of abnormality, cancer staging, and Prostate Imaging Reporting and Data System (PI-RADS) score; PSAD; cancer location and grading (Gleason and International Society of Urological Pathologists (ISUP) grade) on biopsy; Cambridge Prognostic Group (CPG) [10]; and eventual treatment received.

Fisher’s exact test was used to assess the statistical significance (determined to be p<0.05) of the difference between the two groups (aDRE and rPSA) for: biopsy rate after MRI; and combined diagnostic yield of having an MRI and biopsy (if indicated). Diagnostic yield was therefore defined as the percentage of patients who underwent an MRI, who were diagnosed with biopsy-proven cancer.

## Results

Five hundred seventeen patients were triaged onto the BTiPP in 2021. Of these 108 (20.9%) were categorized into the aDRE group, and 398 (77.0%) were in the rPSA group. 11 (2.1%) patients were referred for other reasons and were therefore excluded from any analysis. The mean age of the patients was 68.0 years (range=32 to 96 years, standard deviation=10.6 years).

Thirty-four aDRE patients did not undergo MRI. 27 of them were seen in a clinic by a urologist, for example, for associated lower urinary tract symptoms (LUTS) or haematospermia, and determined to have an unsuspicious DRE. Three aDRE patients were deemed unfit to undergo further investigation or treatment. Three aDRE patients had MRI-incompatible inserted cardiac devices, two of which had a computed tomography (CT) scan to assess the prostate, and one of which proceeded directly to prostate biopsy. One aDRE patient did not have an MRI on the pathway because the case was discussed at the multi-disciplinary team (MDT) meeting, including a recent CT scan of the pelvis, and deemed not concerning for prostate cancer overall.

Therefore, 74 aDRE patients underwent MRI. 41 aDRE patients had MRIs showing no lesion (PI-RADS<3), 11 had PI-RADS=3 lesions, 11 had PI-RADS=4, and 11 had PI-RADS=5. Only one of the patients with a PI-RADS=3 lesion had PSAD>0.15 ng/ml2. Therefore 23 aDRE patients had a biopsy indicated, but one patient was deemed unfit for biopsy and one patient did not consent to biopsy. Therefore 21 patients who had an MRI subsequently had a biopsy; so the biopsy rate after the MRI in the aDRE group was 28.4% (21/74). 15 of those biopsies resulted in cancer diagnosis: therefore, the combined diagnostic yield of MRI and biopsy (if indicated) in the aDRE group was 20.3% (15/74).

A total of 58.1% (43/74) of aDRE patients that underwent MRI had no posterior lesions on MRI. There was one aDRE patient with solely anterior biopsy-proven prostate cancer. This was a Gleason grade 3+4=7, ISUP grade 2, CPG-2 cancer, which was then managed with active surveillance. Therefore, 6.7% (1/15) of biopsy-proven cancers in the aDRE group were solely anterior. There was one aDRE patient with solely anterior disease on MRI but on biopsy had both anterior and posterior cancer. This was Gleason grade 3+4=7, ISUP grade 2, CPG-2 cancer managed with low-dose brachytherapy.

**Figure 1:**
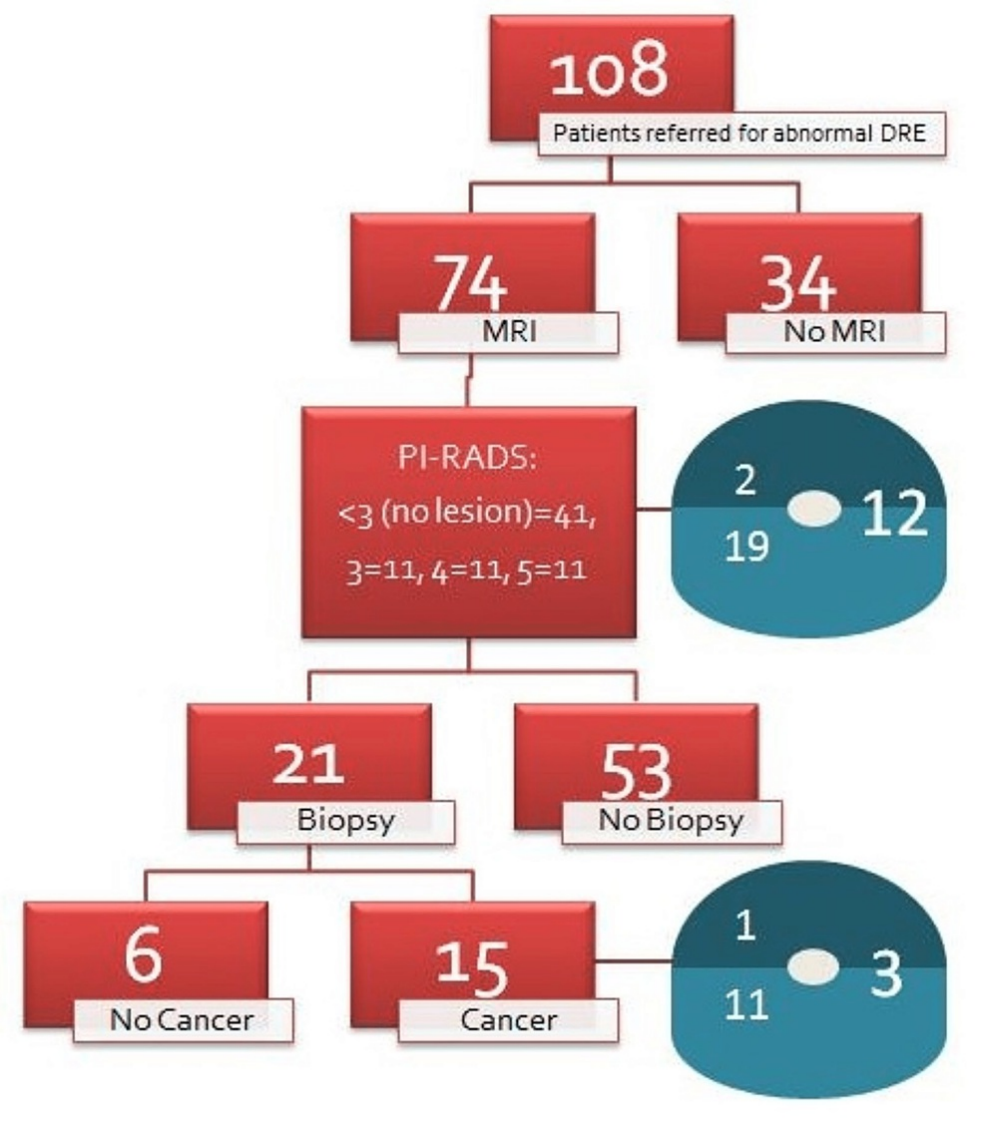
Flowchart showing the pathway of the patients referred for abnormal DRE, and their MRI and biopsy findings. The dark blue indicates abnormality in the anterior prostate only and the light blue indicates abnormality in the posterior prostate only. The larger number indicates the number of patients with both anterior and posterior abnormality. DRE: digital rectal examination; PI-RADS: Prostate Imaging-Reporting and Data System score

Figure 1 shows the pathway of the aDRE patients as well as the location of their abnormalities on MRI and biopsy.

Two hundred eighty-two rPSA patients underwent MRI. 121 of these proceeded to biopsy, so the biopsy rate after MRI in the rPSA group was 42.9% (121/282), which was greater than the 28.4% observed in the aDRE group (p=0.02). 104 of those biopsies resulted in cancer diagnosis: therefore, the combined diagnostic yield of MRI and biopsy (if indicated) in the rPSA group was 36.9% (104/282), which was greater than the 20.3% in the aDRE group (p=<0.01).

None of the 11 patients referred for other reasons underwent an MRI or biopsy.

Table 1 outlines the results for aDRE and rPSA groups.

**Table 1:** Number of patients in both groups, undergoing MRI, biopsy after MRI, and biopsy-proven cancers. aDRE: abnormal DRE group (referred for abnormal DRE with normal PSA); rPSA: raised PSA group (referred for raised PSA regardless of DRE findings).

Number of Patients	aDRE	rPSA	p-value (Fisher’s exact test)
Total	108	398	
Underwent MRI	74	282	
Biopsy after MRI (Biopsy rate)	21 (28.4%)	121 (42.9%)	0.02
Biopsy-proven cancer after MRI (Combined diagnostic yield)	15 (20.3%)	104 (36.9%)	<0.01

## Discussion

This study directly compared the biopsy rate after MRI and combined diagnostic yield of MRI and biopsy (if necessary) between patients referred for abnormal DRE (with normal PSA) and those referred for raised PSA. It is the first study of its kind and therefore difficult to correlate with existing literature. However this real-world comparison is a highly pragmatic one when considering a change to the diagnostic pathway. These results should be considered by other centers utilizing similar practice.

Biopsy rate and combined diagnostic yield were both significantly lower in the aDRE group compared with the rPSA group. The BTiPP recommends up-front MRI for patients referred for either abnormal DRE or raised PSA [1], but these results call into question whether aDRE patients require an adjusted pathway. The findings of this study are in keeping with the previously mentioned study showing that significant cancer detection rate was higher with a raised PSA than with normal PSA for patients with an abnormal DRE [7]. This study had a smaller sample size than the work presented here but also made a different comparison from a pragmatic point of view. The authors also found that concordance between primary and secondary care DRE findings was only 46% [7]. This adds to the rationale of the work presented here, along with the previously mentioned studies demonstrating the poor sensitivity of DRE [3] and lack of confidence in DRE in primary care [4].

Although lower than in rPSA patients, the combined diagnostic yield of 20.3% for aDRE patients in our study is still significant. Furthermore, two large studies found that abnormal DRE was a stronger predictor of prostate cancer at higher PSA levels [5, 6], indicating that the diagnostic yield of all the patients referred with abnormal DRE (with raised or normal PSA) may have been even higher in our cohort. These points emphasize the importance of DRE in primary care when considering specialty referral, especially as DRE is often performed prior to the acquisition of a PSA result, and the need for rapid assessment in the investigation of possible cancer.

A large case-control study of patients who underwent radical prostatectomy found that anterior-dominant prostate cancers have significantly lower rates of abnormal DRE findings compared to posterior cancers (10% vs. 23%) [11]. We found that 58.1% of aDRE patients who underwent MRI had no lesions in the posterior prostate gland, where lesions would most likely be detected on DRE. This could indicate that many of the cancers in aDRE patients were detected incidentally rather than having been palpated on examination, particularly the aDRE patient with solely anterior disease.

To summarize, DRE is a valuable tool alongside PSA for prostate cancer investigation, however, an abnormal DRE performed in primary care with normal PSA is less likely to indicate prostate cancer than with a raised PSA. Further work is required to contribute to the formation of a consensus on the role of DRE in prostate cancer investigation.

Our results suggest that adopting an adjusted pathway whereby aDRE patients are reviewed in a clinic for DRE by a urologist could reduce the number of MRIs performed. In 2021 at our institution, this would have required 108 additional outpatient appointments. Assuming MRI-proven posterior prostate lesions are palpable and anterior lesions are impalpable, this adjusted pathway could have prevented 43 MRI scans in patients where the MRI eventually showed no significant posterior lesion (PI-RADS <3). This would represent a 12.1% (43/356) reduction in the annual MRI prostate burden at our institution. The change would also free up slots for biopsies and MRI scans for other specialties. It would also prevent some patients from undergoing unnecessary biopsies and treatments, avoiding complications from these and patient anxiety. The adjusted pathway has therefore been considered by the urology department at our institution, a consensus was reached, and the change has been implemented.

There would be financial costs associated with the additional consultant face-to-face appointments required for the adjusted pathway, but this would likely be offset by the savings from performing fewer MRI scans and biopsies. One limitation of this change would be potential delays to the investigation and treatment of some prostate cancers. On the adjusted pathway the 2/108 (1.8%) patients with solely anterior lesions on MRI may not have had their cancer detected on DRE, so may have been missed, which represents another potential limitation to this change. However, one of them had biopsy-proven cancer in the anterior and posterior cores, which may have therefore been detected on DRE by a urologist. Furthermore, both these patients were categorized as CPG-2, which has a favorable prognosis [10]. One of these patients was managed with active surveillance and one had low-dose brachytherapy. Therefore, it may be reasonable to accept that 1.8% of aDRE patients on the adjusted pathway could have their cancer missed, given the fact that they were low-grade and may not require active treatment. Prostate cancer patients with PSA<3.0 ng/ml are more likely to have a favourable prognosis, and very unlikely to have advanced cancer [6]. Whether a patient would like to be further investigated for a clinically insignificant prostate cancer will vary depending on the individual, but given the significant risks of biopsies and treatment, it is important to define a clear cut-off for when further investigation is not in a patient’s best interests. It is likely that the pathway modification proposed here will result in fewer diagnoses of clinically insignificant prostate cancers.

The limitations of this study are that it is a retrospective study in a single center and that no power calculation was performed. Its strengths include its large sample size compared to similar work [7]. It is also the first study to directly compare the biopsy rate after MRI and combined diagnostic yield of MRI and biopsy (if necessary) between patients referred for abnormal DRE alone (with normal PSA) and those referred with raised PSA.

## Conclusions

Following MRI, the biopsy rate and diagnostic yield were both significantly lower in the aDRE group compared with the rPSA group. We propose a modification to the BTiPP so that patients referred for abnormal DRE with normal PSA are seen in a clinic by a urologist before MRI, in order to prevent unnecessary investigations, treatment, and patient anxiety. These conclusions have led to such a modification to the diagnostic pathway at our institution, and we propose that this be considered by other institutions in the UK and beyond.
